# Gut Microbial Glycerol Metabolism as an Endogenous Acrolein Source

**DOI:** 10.1128/mBio.01947-17

**Published:** 2018-01-16

**Authors:** Jianbo Zhang, Shana Sturla, Christophe Lacroix, Clarissa Schwab

**Affiliations:** a Department of Health Sciences and Technology, ETH Zürich, Zürich, Switzerland; University of Wisconsin—Madison

**Keywords:** endogenous acrolein, glycerol metabolism, gut microbiota, reuterin, toxicity

## Abstract

Acrolein is a highly reactive electrophile causing toxic effects, such as DNA and protein adduction, oxidative stress, endoplasmic reticulum stress, immune dysfunction, and membrane damage. This Opinion/Hypothesis provides an overview of endogenous and exogenous acrolein sources, acrolein’s mode of action, and its metabolic fate. Recent reports underpin the finding that gut microbial glycerol metabolism leading to the formation of reuterin is an additional source of endogenous acrolein. Reuterin is an antimicrobial multicomponent system consisting of 3-hydroxypropionaldehyde, its dimer and hydrate, and also acrolein. The major conclusion is that gut microbes can metabolize glycerol to reuterin and that this transformation occurs *in vivo*. Given the known toxicity of acrolein, the observation that acrolein is formed in the gut necessitates further investigations on functional relevance for gut microbiota and the host.

## EXPOSURE, TOXICITY, AND FATE OF ACROLEIN

### Human exposure to acrolein.

Acrolein, 2-propenal, is the simplest *α*,*β*-unsaturated aldehyde and a ubiquitous contaminant found in the environment and food. Exogenous sources of acrolein include tobacco smoke, exhaust gas emission, wood combustion, and deep-fat frying (1). As a result, acrolein can be detected in air, surface water, and various kinds of food. For example, 6.9 to 29.8 µg m^−3^ acrolein has been detected in the indoor air in restaurants in Germany (1). In addition, acrolein was recovered from food and beverages such as whisky (0.7 to 11.1 µg liter^−1^) and red wine (3,800 µg liter^−1^) (1). Levels reported for alcoholic beverages exceed minimal risk levels (4 µg kg^−1^ body weight day^−1^ for intermediate-duration oral exposure) (2) and the chronic oral dose (0.05 µg kg^−1^ body weight day^−1^) (2).

Endogenous formation of acrolein by chemical reactions and mammalian enzymatic activity has been investigated in depth (3). Myeloperoxidase, a heme enzyme excreted by human neutrophils, converts hydroxy-amino acids, e.g., threonine, to acrolein in the presence of H_2_O_2_ and a chlorine ion (Table 1). Acrolein can also be produced by copper-dependent amine oxidation of spermidine and spermine, followed by spontaneous retro-Michael-type cleavage (3). In addition, catabolism of oxazaphosphorine drugs, such as cyclophosphamide, produces acrolein (4). Moreover, Uchida et al. described lipid peroxidation as an important source of acrolein (5). While exposure to exogenous acrolein sources might be transient, acrolein can constantly be formed endogenously, raising the concern about its chronic toxicity.

**TABLE 1:** Overview of endogenous sources of acrolein

Precursor(s)	Class(es)	Key mechanism(s)	Reference
Threonine	Amino acids	Myeloperoxidase, H_2_O_2_, Cl^−1^	3
Spermidine	Polyamines	Amine oxidase, retro-Michael-type cleavage	3
Polyunsaturated acids	Lipoproteins/lipids	Peroxidation	5
Cyclophosphamide	Anticancer drugs	Oxidative-ring opening	4

### Mechanisms of acrolein toxicity.

Acrolein is a highly reactive electrophile that modifies cellular nucleophiles, giving rise to adverse responses involving multiple molecular mechanisms (Table 2). Acrolein can form cyclic DNA adducts by addition to the 1 and N^2^ positions of deoxyguanosine (6). Frequency and distribution of these acrolein-DNA adducts along the tumor suppressor gene *p53* in human bronchial epithelial cells matched *p53* mutations in cigarette smoking-related lung cancer (7). Binding of acrolein to amines of amino acid residues can lead to protein dysfunction. Uchida et al. found that lysine and histidine residues of low-density lipoprotein (LDL) can be modified by acrolein by covalent binding (5). Using *N*^α^-acetyl-lysine and *N*^α^-acetyl-histidine as model molecules, *N*^α^-acetyl-*N*^*ε*^-(3-formyl-3,4-dihydropyridine)-lysine and *N*^α^-acetyl-*N*^im^-propanalhistidine were identified as the major adducts. These adducts may contribute to the dysfunction of the antiatherogenic apolipoprotein E (8). Reaction of acrolein with cysteine thiols of proteins leads to the formation of beta-propanal adducts through Michael addition, which can inactivate important enzymes. Besides having direct effects on biomolecular function from covalent modification of DNA or proteins, acrolein can induce indirect toxic effects by disrupting various signaling pathways. Acrolein induces apoptosis, endoplasmic reticulum stress, and oxidative stresses (for a review, see reference 9). Acrolein can decrease mitochondrial membrane potential and active apoptotic enzymes, such as caspase 9 and caspase 7 (9). Acrolein can trigger immune and inflammatory responses, such as the increased expression of nuclear factor kappa B (NF-κB), tumor necrosis factor alpha (TNF-α), interleukin 6 (IL-6), or IL-8, contributing to endoplasmic reticulum stress (9). Moreover, acrolein decreased barrier function and increased permeability, potentially due to the downregulation of tight junction proteins ZO-1, occludin, and claudin-1 (10). Together, these mechanisms of acrolein toxicity contribute to the pathogenesis of various diseases and xenobiotic intoxication.

**TABLE 2:** Targets and modes of action of acrolein and their consequences

Effects	Mode of action	Molecular event(s)^a^	Reference
Direct			
DNA mutation	DNA adducts	Conjugation of DNA bases	7
Protein dysfunction	Amino acid adducts	Conjugation of amino acids bearing an amine/thiol group	8
Indirect			
Apoptosis	Mitochondrial dysfunction	Mitochondrial membrane potential ↓, caspase 7/9↑, caspase 3↓	9
Endoplasmic reticulum stress	Immune and inflammatory responses	NF-κB ↑, TNF-α ↑, IL-6 ↑, IL-8 ↑	9
Intestinal barrier dysfunction	Downregulation of tight junction proteins	ZO-1 ↓, occludin ↓, claudin-1 ↓	10

a Arrows represent upregulation (↑) and downregulation (↓).

### Fate of acrolein.

The metabolism, distribution, and excretion of acrolein *in vivo* has been well characterized. In rats administered radiolabeled acrolein (2.5 mg kg^−1^ body weight) by oral gavage, urinary excretion, carbon dioxide expiration, and fecal excretion was 52 to 63%, 30 to 31%, and 13 to 15%, respectively. Residual radioactivity in tissue was minimal (<1.2%) (11). Similar excretion patterns were observed in rats dosed intravenously, with higher urinary excretion and CO_2_ expiration and lower fecal excretion (11). These observations suggest that acrolein was quickly taken up, metabolized, and excreted. The major metabolic pathway of acrolein is conjugation with glutathione (GSH), followed by enzymatic cleavage of γ-glutamic acid and glycine and acetylation of the resulting cysteine adduct, giving rise to *S*-(3-oxopropyl)-*N*-acetylcysteine (OMPA). Oxidation of OMPA produces carboxylethylmercapturic acid (CEMA). Minor products of direct acrolein metabolism include glyceraldehyde, oxalic acid, malonic acid, and 3-hydroxypropionic acid (3). Reduction of OMPA produces 3-hydroxylpropylmercapturic acid (3-HPMA), which is the major acrolein-derived metabolite in urine (3). 3-HPMA has been used as a biomarker of human exposure to tobacco smoking-derived acrolein (12). Intriguingly, urine samples of nonsmokers also contain 3-HPMA (12), suggesting that humans are constantly exposed to endogenous acrolein.

## MICROBIAL GLYCEROL METABOLISM AND THE REUTERIN SYSTEM

### Bacterial GDH reduces glycerol to 3-HPA, a key component of reuterin.

**FIG 1:**
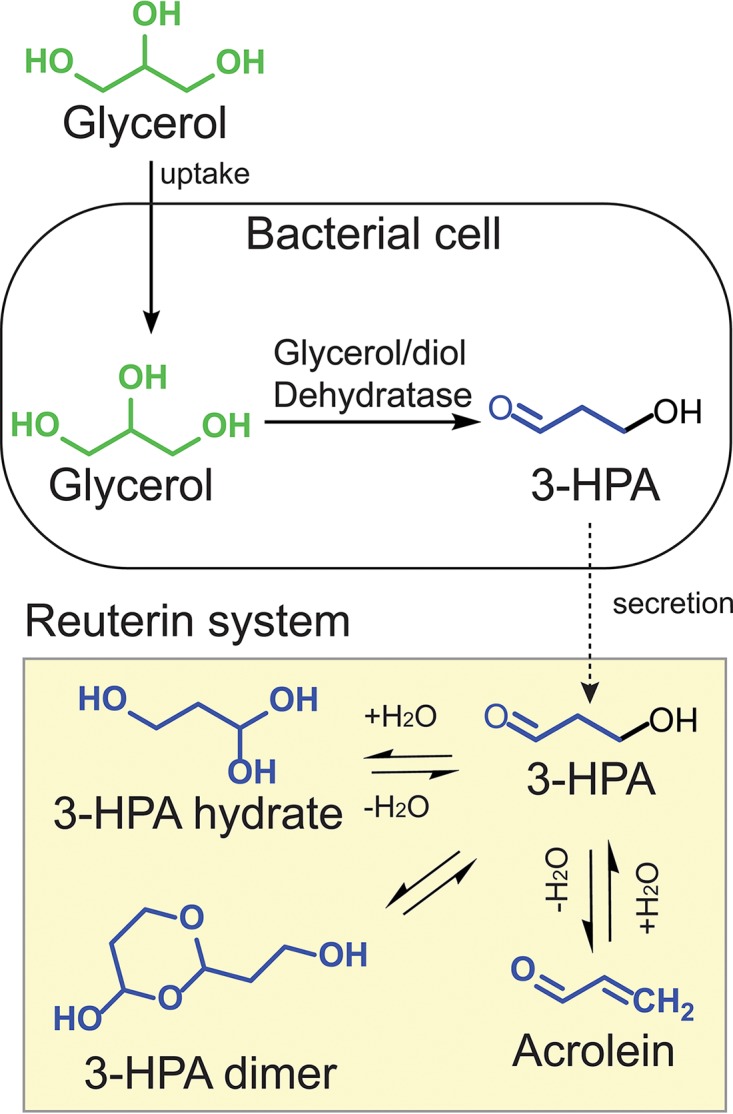
Conversion of glycerol to 3-hydroxypropionaldehyde (3-HPA) by bacterial glycerol/diol dehydratases (GDH) and major components of the reuterin system.

The human gut microbiota may be an as-yet-unrecognized source of endogenous acrolein. Using a combined analytic approach, we recently showed that acrolein is a product of bacterial glycerol metabolism (13). Bacterial vitamin B_12_-dependent glycerol/diol dehydratases (GDH) reduce glycerol to 3-hydroxypropionaldehyde (3-HPA) (13). A second substrate for these enzymes is 1,2-propanediol (1,2-PD), which is reduced to propanal, an intermediate of propionate formation (14). In aqueous solution, 3-HPA exists in an equilibrium with mainly its hydrate, 1,1,3-propanetriol, and its dimer, 2-(2-hydroxyethyl)-4-hydroxy-1,3-dioxane (13). This dynamic system (Fig. 1) has been called reuterin after *Lactobacillus reuteri*, the best-studied reuterin producer. 3-HPA also spontaneously dehydrates to acrolein, and at physiological conditions (pH 7, 37°C), acrolein is always present in solution, including in microbiological fermentation broth (13). These data suggest that acrolein should be considered an intrinsic component of the reuterin system (13).

### Contribution of acrolein to antimicrobial activity and chemical reactions attributed to reuterin.

Reuterin exhibits inhibitory activity against a broad range of Gram-positive and Gram-negative bacteria, yeasts, molds, and protozoa (15). Conjugation of acrolein with GSH may cause depletion of thiol pools in cells, which is believed to contribute to the antimicrobial activity of reuterin (13). In addition to having an antimicrobial function, reuterin is implicated in the conjugation of food-derived carcinogenic heterocyclic amines (HCA), a process of potential relevance to the availability and carcinogenicity of HCA in the human gut (16–18). The HCA 2-amino-1-methyl-6-phenylimidazo[4,5-b]pyridine (PhIP), an amino acid pyrolysis product formed when meat is cooked at high temperatures, is transformed to a glycerol conjugate metabolite, 7-hydroxy-5-methyl-3-phenyl-6,7,8,9-tetrahydropyrido[3′,2′:4,5]imidazo[1,2-α]pyrimidine-5-ium chloride (PhIP-M1) in the presence of *L. reuteri* or enterococci (16). Using the acrolein scavengers GSH and *N*-acetyl-l-cysteine (or PhIP as a reactive probe for acrolein), we showed that acrolein is the active compound of the reuterin system with regard to antimicrobial activity and PhIP transformation (13).

### Human gut microbes produce reuterin.

Eleven percent of gut microbes are predicted to possess vitamin B_12_-dependent GDH and are therefore likely able to produce 3-HPA from glycerol (19). Reuterin-forming *L. reuteri* organisms have been isolated from human feces but occur in only low abundance in some humans (14). Screening fecal metagenomes of adult healthy humans, putative GDH-encoding genes were detected in members of the phyla *Firmicutes* (*Eubacterium hallii*, *Blautia obeum*, *Ruminococcus gnavus*, *Flavonifractor plautii*, *Intestinimonas butyriciproducen*s, and *Veillonella* spp.) and *Proteobacteria* (*Escherichia coli*, *Klebsiella* spp., and *Citrobacter* spp.), indicating functional redundancy across phylogenetically different taxons (14). We used *E. hallii* as a gut-derived model organism to verify the predictions made by metagenome and genome analyses (the presence of a glycerol/diol dehydratase and vitamin B_12_ biosynthesis genes) in microbiological assays (14). These assays confirmed vitamin B_12_ synthesis and the formation of 3-HPA and propanal from glycerol and 1,2-PD, respectively, with propanal being further metabolized to propanol and propionate (14). In growing cultures of *L. reuteri*, a major proportion of 3-HPA is reduced to 1,3-propanediol (1,3-PD) by a NAD^+^-dependent oxidoreductase, allowing cofactor regeneration (20). *E. hallii* does not form 1,3-PD, which might lead to the accumulation of 3-HPA and acrolein and, ultimately, to the transformation of PhIP to PhIP-M1 (17). PhIP-M1 was also recovered from *L. reuteri* grown in the presence of glycerol despite a major proportion of 3-HPA being further metabolized to 1,3-PD, indicating that acrolein was released. Further gut microbes with predicted glycerol/diol dehydrates were shown to form 3-HPA from glycerol. *Klebsiella* and *Citrobacter* species produced 3-HPA during growth in the presence of glycerol in addition to 1,3-PD (21), and the ability of *R. gnavus* to form propionate from 1,2-propanediol (22) implies the presence of an active GDH and the potential of this species to metabolize glycerol to 3-HPA.

### Bacterial formation of acrolein in the human intestine.

**FIG 2:**
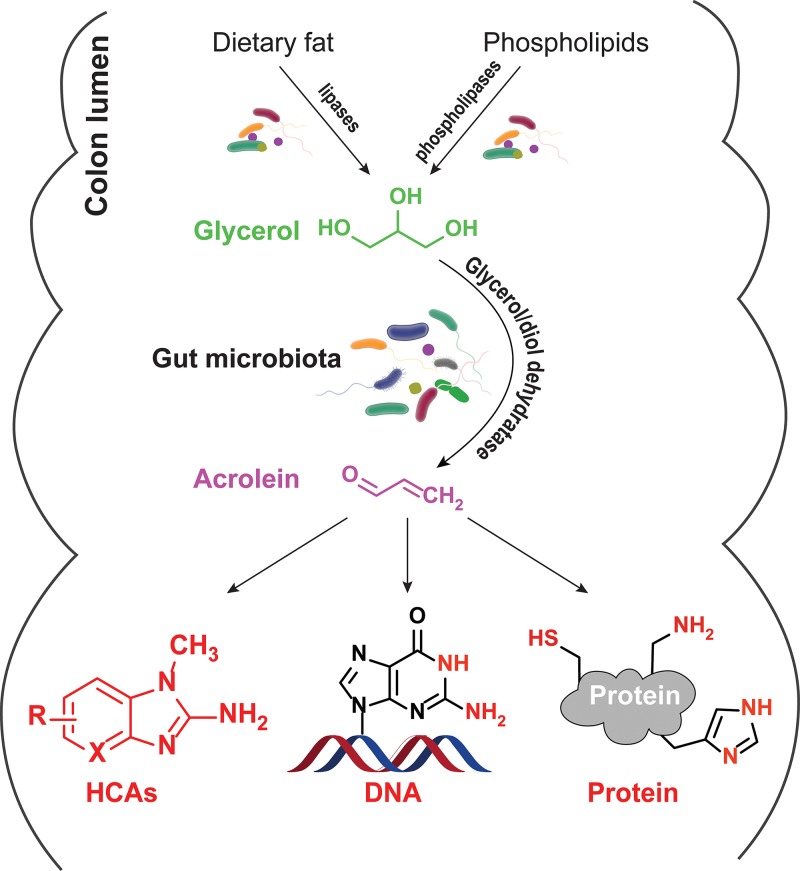
Overview of the endogenous precursors, formation, and potential targets of acrolein in the gut.

If there is any physiological relevance for the conversion of glycerol to acrolein in the human gut, it is essential that glycerol be present and that GDH be expressed by the human gut microbiota. Glycerol is a common additive in formulated foods, where it is used as a sweetener, humectant, and moisturizing or thickening agent, and glycerol can be liberated from tri-, di-, and mono-glycerides by digestive lipases in the small intestine (23). While glycerol is likely well absorbed in the small intestine, limited saturation of this process (24) leads to a portion of the chemical reaching the colon. Moreover, bacterial lipases of, for example, *Prevotella intermedia* (previously *Bacteroides intermedius*), *Fusobacterium necrophorum*, and *Eubacterium combesii* are active in the colon (Fig. 2) (23). Additionally, bacteria expressing phospholipases can hydrolyze phospholipids from the cell membrane to produce glycerol (Fig. 2). Thus, glycerol has been observed in human feces (25).

An anticipated fate of intestinal glycerol is its reduction to 3-HPA, based on the common presence of genes encoding GDH in metagenomes and its conversion to acrolein, supported by the observation of acrolein transformation products. The heterocyclic amines PhIP and MeIQx (2-amino-3,8-dimethylimidazo[4,5-*f*]quinoxaline) are converted to PhIP-M1 and MelQx-M1 by complex colonic microbiota in the presence of glycerol (17, 18). Vanhaecke and coworkers reported that PhIP was transformed to PhIP-M1 by 18 fecal microbiota from individual donors, with PhIP transformation efficiencies ranging from 1.8% to 96% (16). Using an *in vitro* continuous fermentation model, PolyFermS, it could be shown that inactive microbiota can be made to significantly promote HCA transformation by addition of a reuterin-producing, GDH-bearing strain of *E. hallii* (17, 18). Finally, PhIP-M1 could be recovered from feces of consumers that obtained a single portion of cooked chicken meat containing PhIP (26). Variations in the occurrence and abundances of gut microbes with GDH among individuals and further metabolism of reuterin to 1,3-PD by some strains (20) might be a reason for interindividual variations in acrolein formation-respective HCA degradation proﬁciency and for susceptibility to the development of colorectal cancer.

## CONCLUSIONS

The assertion that acrolein is produced from microbial glycerol metabolism in the human gut (Fig. 2) is supported by several strong points of evidence: (i) dietary HCAs are converted to acrolein conjugates by fecal and colon microbiota when glycerol is present (16, 17), (ii) a substantial proportion of 3-HPA is converted to acrolein under conditions prevailing in the human colon (13), and (iii) glycerol is present in the colon. Such production of acrolein in the human gut lumen may be regarded as a double-edged sword with regard to toxicological relevance. On one hand, acrolein conjugation of HCAs appears to be a detoxification process (18, 26), suggesting that microbially produced acrolein might attenuate carcinogenesis, but on the other hand, acrolein itself is toxic. This situation raises the question of whether chronic exposure to acrolein formed in the gut lumen by microbial metabolism has a net adverse influence on health or contributes in any beneficial manner. Moreover, since *L. reuteri* strains that are used as probiotics possess *gdh* and form reuterin and, potentially, acrolein, it may be prudent to reevaluate the safety of probiotic use of *L. reuteri*. Acrolein also is a broad-spectrum antimicrobial. However, to predict the antimicrobial impact of a highly reactive component, such as acrolein, may be very difficult in a complex ecosystem, such as the gut.

While further research is needed to define the physiological implications of acrolein for the gut microbiota and the host, gut microbial glycerol metabolism should be considered a relevant endogenous source of acrolein.
